# An analysis of the complementarity of ICECAP-A and EQ-5D-3 L in an adult population of patients with knee pain

**DOI:** 10.1186/s12955-016-0430-x

**Published:** 2016-03-03

**Authors:** T. Keeley, J. Coast, E. Nicholls, N. E. Foster, S. Jowett, H. Al-Janabi

**Affiliations:** MRC Midland Hub for Trials Methodology Research, University of Birmingham, Edgbaston, Birmingham, B15 2TT UK; Health Economics Unit, Institute of Applied Health Research, Public Health Building, University of Birmingham, Edgbaston, Birmingham, B15 2TT UK; Arthritis Research UK Primary Care Centre, Research Institute for Primary Care and Health Sciences, Keele University, Keele Staffordshire, ST5 5BG UK; School of Social and Community Medicine, University of Bristol, Bristol, BS8 2PS UK

## Abstract

**Background:**

The ICECAP measures potentially offer a broader assessment of quality of life and well-being, in comparison to measures routinely used in economic evaluation, such as the EQ-5D-3 L. This broader assessment may allow measurement of the full effects of an intervention or treatment. Previous research has indicated that the ICECAP-O (for older people) and EQ-5D-3 L measure provide complementary information. This paper aims to determine similar information for the ICECAP-A (for the entire adult population) in terms of whether the measure is a substitute or complement to the EQ-5D-3 L.

**Methods:**

Data from the BEEP trial - a multi-centre, pragmatic, randomised controlled trial - were used. Spearman rank correlations and exploratory factor analytic methods were used to assess whether ICECAP-A and EQ-5D-3 L are measuring the same, or different, constructs.

**Results:**

A correlation of 0.49 (*p* < 0.01) was found between the ICECAP-A tariff score and the EQ-5D-3 L index. Using the pooled items of the EQ-5D-3 L and the ICECAP-A a two factor solution was optimal, with the majority of EQ-5D-3 L items loading onto one factor and the majority of ICECAP-A items onto another.

**Conclusion:**

The results presented in this paper indicate that ICECAP-A and EQ-5D-3 L are measuring two different constructs and provide largely different, complementary information. Results showed a similarity to results presented by Davis et al. using the ICECAP-O.

**Trial registration:**

ISRCTN 93634563

## Background

The ICECAP-A and the ICECAP-O are two relatively new patient reported outcome measures of wellbeing. Both measures have value weighted tariffs attached, so may be appropriate for use in economic evaluation, as well as in evaluations of the effectiveness of an intervention. These measures have their theoretical underpinnings in Amartya Sen’s work on functioning and capability [1], which advocates an assessment of wellbeing that maintains a focus on what a person is able to do (capability), rather than what a person does (functioning). The capability approach encourages a broad evaluative space which can include a person’s ability to achieve their basic requirements, such as living in good health, and more complex abilities, such as the ability to achieve things that are important to them, like fulfilling social or professional roles [1].

**Fig. 1:**
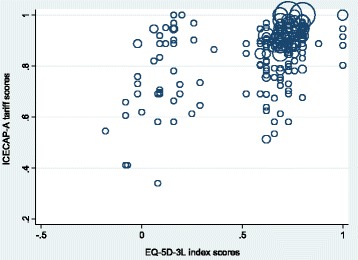
ICECAP-A and EQ-5D scores, data points weighted by frequency count

The ICECAP-A is intended for use with adults, with a sister measure (the ICECAP-O) available for use with older people. The ICECAP-A measures capability in Attachment, Stability, Achievement, Enjoyment and Autonomy [2] (while the ICECAP-O measures capability in Attachment, Security, Role, Enjoyment and Control [3]). A score of one on both ICECAP measures indicates full capability and the measures are anchored to “no capability” indicated by a score of 0. Scores can be used in economic evaluations through the use of full capability [4] or sufficient capability [5] approaches.

The most commonly used measure within economic evaluations, in contrast, is a generic preference based outcome focusing on health-related quality of life [6]. The descriptive system for this measure, the EQ-5D-3 L, comprises mobility, self-care, usual activities, pain and discomfort, and anxiety and depression [7]. The three level version, the EQ-5D-3 L has been extensively validated in numerous clinical settings [8–11]. A score of one indicates full health and states worse than death are represented by scores between zero and−0.59. Scores from the EQ-5D-3 L are used in economic evaluation through the calculation of the quality-adjusted life year [12]. The five level version of the EQ-5D was not available for use at the start of this trial.

The broader assessment of quality of life potentially offered by the ICECAP-A may allow researchers to assess changes in attributes that are not routinely evaluated [13–15]. The ICECAP-A is being used alongside the EQ-5D-3 L in a number of studies [16–18]. If the ICECAP-A assesses additional attributes not measured by the EQ-5D-3 L, then a strong case can be made for using both measures in tandem (especially if the intervention being tested targets attributes measured by the ICECAP-A). However, if the ICECAP-A provides little additional information, then use of multiple instruments in research studies may be counter-productive, as it will increase patient burden, increase the potential for selective reporting [19, 20] and thus may lead to problems in synthesis of evidence at later stages [21]. Determining which of these potential situations is the case for ICECAP-A is therefore important, and provides the focus for this research. The results will help to determine the additional benefit of including the ICECAP-A in empirical work, including economic evaluation.

Earlier work, using exploratory factor analysis, determined that the ICECAP-O and EQ-5D-3 L provided “largely unique and complementary information so are not substitutes” _p.975_ [22]. Results indicated that the ICECAP-O items of Attachment, Security, Role and Enjoyment and the EQ-5D-3 L item of anxiety/depression represented a single factor which the authors termed “psychological well-being”. The EQ-5D-3 L items of mobility, self-care, usual activities and pain represented a single factor termed “physical functioning”. While they are similar measures, it cannot be assumed these results for the ICECAP-O will hold for the ICECAP-A. Using factor analytic methods, which are similar to the methodology used by Davis et al. [22], we aim to assess whether the ICECAP-A measure is a complement or substitute for the EQ-5D-3 L.

## Methods

This analysis was completed using data from the Benefits of Effective Exercise for knee Pain (BEEP) trial, a primary care, multi-centre, pragmatic randomised controlled trial in the UK. This trial aimed to compare improvement in pain and function outcomes from three physiotherapy-led exercise interventions for older adults with knee pain attributable to osteoarthritis [23]. The ICECAP-A and EQ-5D-3 L were administered and baseline data are used in this analysis.

### Association

Spearman’s rank correlation was used to assess association between the ICECAP-A tariff scores [24] and the index scores of the EQ-5D-3.

### Exploratory factor analysis

Exploratory factor analysis is a statistical technique based on the premise that a battery of questions can be described based on a smaller number of underlying factors. Factor analysis describes variability amongst a number of variables or items through the use of a smaller number of unobserved variables, known as factors [25]. If a scale is uni-dimensional then one factor should explain the variance accurately [25]. Factor analysis can also be used to test the assumption that a pool of items assesses different underlying factors.

Exploratory factor analysis assumes that variables are continuous and follow a normal distribution. When using categorical variables, exploratory factor analysis can be performed using polychoric correlations, which are suitable for categorical variables or variables that do not follow normal distribution. The number of factors retained was chosen with reference to the Kaiser Criterion [26], which advocates retaining factors with Eigen Values greater than one and using the scree plot to assess the suitability of this choice. An oblique Promax rotation was used, which allows for the potential that factors are correlated. Correlations between factors equal to or greater than 0.32 is considered the point at which oblique rotations are appropriate [27]. Exploratory factor analysis was applied to all items from both the EQ-5D-3 L (5 items) and the ICECAP-A (5 items).

## Results

The characteristics of the BEEP trial participants used in this analysis are presented in Table 1. The mean age of participants was 63, with a roughly equal proportion of male and female participants. The average ICECAP-A capability tariff values were higher (indicating higher capability) at baseline than values previously reported in the general population [28]. Participants reported mean EQ-5D-3 L scores at baseline that were lower than the UK national average for this age group, indicating poorer health-related quality-of-life [6] Other outcome measures indicated that this was a population with low levels of anxiety and depression and moderate levels of pain and physical disability. A complete case analysis was performed, which included all participants who completed both the ICECAP-A and EQ-5D measures at baseline. The ICECAP-A had a 99 % completion rate and the EQ-5D-3 L has a 97 % completion rate, resulting in this factor analysis being completed using 442 participants.

**Table 1:** baseline characteristics

Characteristic	Mean values (SD)	Median values (IQR)	Measure range	Sample range	Sample size
Socio-demographic					
Age (SD)	63.3 (9.9)			45 to 90	456
Gender (% male)	50.2 %^a^				456
Health and functioning					
ICECAP-A tariff	0.88 (0.12)	0.91 (0.85, 0.97	0.0 to 1.0	0.34 to 1.0	452
EQ-5D-3 L index	0.63 (0.24)	0.69 (0.62, 0.76)	−0.59 to 1.0	−0.18 to 1.0	442
WOMAC pain^b^	8.5 (3.5)	8 (6, 11)	0 to 20	0 to 18	449
WOMAC stiffness	3.8 (1.7)	4 (3, 5)	0 to 8	0 to 8	451
WOMAC functioning	28.7 (12.2)	27 (20, 37)	0 to 68	0 to 62	446
GAD-7^c^	3.4 (4.7)	1 (0, 4)	0 to 21	0 to 21	439
PHQ-8^d^	4.1 (4.8)	2 (1, 5)	0 to 24	0 to 24	442

^a^Not a mean value. ^b^WOMAC (Western Ontario and McMaster Universities Osteoarthritis Index). ^c^GAD-7 (General Anxiety Disorder-7) is a measure of anxiety. ^d^PHQ-9 (Patient Health Questionnaire-9) is a measure of depression

### Correlation

A moderate correlation of 0.49 (*p* < 0.01) was found between the ICECAP-A tariff score and the EQ-5D index. The scree plot showed a cluster of participants scoring above 0.6 on the EQ-5D and above 0.8 on the ICECAP-A (Fig. 1).

### Exploratory factor analysis

The results of the exploratory factor analysis are presented in Table 2. Through consideration of a scree plot and the number of Eigen Values greater than one, a two factor solution was found to be optimal. The choice of an oblique promax rotation was explored through use of the STATA “estat common” command (post rotation). This indicated a correlation of−0.52 between the factors, indicating that an oblique promax rotation was an appropriate choice for the analysis.

**Table 2:** Exploratory factor analysis comparing the ICECAP-A and EQ-5D-3 L items (*n* = 442)

	Rotated factor loadings	
	Factor 1	Factor 2
EQ-5D-3 L		
Mobility		0.82
Self-Care		0.79
Usual Activities		0.69
Pain		0.67
Anxiety and Depression	−0.74	
ICECAP-A		
Stability	0.86	
Attachment	0.67	
Autonomy	0.40	−0.46
Achievement	0.68	−0.22
Enjoyment	0.83	
Between factor correlation	−0.52	

Loadings of <0.2 are dropped from the table to allow easy interpretation of results

Table 2 shows a two factor solution indicating that two separate, but correlated factors are assessed by the pooled items of EQ-5D-3 L and the ICECAP-A. The majority of EQ-5D-3 L items (Mobility, Self-care, Usual Activities and Pain) loaded strongly onto factor two, while the majority of ICECAP-A items (Stability, Attachment, Achievement, Enjoyment) loaded onto factor one. The EQ-5D-3 L item of Anxiety and Depression loaded strongly onto factor one and the loading of Autonomy split, with moderate loadings onto both factors.

## Discussion

The results presented in this paper indicate that the ICECAP-A and EQ-5D-3 L are measuring two different constructs and therefore provide largely different information. Results showed a strong similarity to results presented by Davis et al. [22] using the ICECAP-O.

The two factor solution found that the majority of ICECAP-A items loaded onto one factor and the majority of EQ-5D-3 L items onto another. The exception to this was the EQ-5D-3 L item of anxiety and depression, which loaded onto a factor with Stability, Attachment, Achievement and Enjoyment. The Autonomy item loaded moderately onto both factors. The Autonomy item assesses a similar attribute to the Control item in the ICECAP-O, which Davis et al. found to split between the two factors.

This analysis was completed in an older population with higher capability scores than the general public, with worse than average health states, therefore caution should be exercised when generalising these results to the general population, other patient groups or groups of different ages. However, the analysis is consistent with previous work on the ICECAP-O and EQ-5D-3 L which also suggested that the ICECAP-O and EQ-5D-3 L are measuring two separate, but correlated factors [22]. A further limitation was the lack of availability of the EQ-5D-5 L at the start of the BEEP trial. However, the descriptive systems of both the three and five level measures comprise the same five dimensions and considering the results of the EQ-5D crosswalk study [29], one may cautiously expect similar results using the EQ-5D-5 L.

Davis et al. have previously suggested that these factors be termed “physical functioning” and “psychosocial wellbeing”. We suggest that “physical health” and “wellbeing” may be more reflective of how these constructs are normally termed in health economic evaluation and in academic literature. This change in terminology from that suggested by Davis et al. was based on three lines of thought. First, the EQ-5D and other similar measures are conceptually focused on measuring health gain (rather than functioning), as the primary focus of economic evaluation. The findings of this work and to a large extent the Davis work was that the physical aspects of EQ-5D loaded onto one factor, hence physical health. Second, functioning as a term has a specific meaning in relation to the capability approach (which classifies outcomes in terms of functionings and capabilities, related to whether people do these things or are just able to do them; EQ-5D expresses one of its dimensions as ability to conduct usual activities and hence to refer to this as a functioning is potentially confusing from a capability perspective) and using it in this way could cause conceptual confusion. Third, it is not clear that all aspects captured by the second factor are ‘psychosocial’ particularly in relation to achievement and autonomy (the latter of which loaded onto both factors); wellbeing, as a broader concept, seems to more accurately capture the nature of the totality of attributes included in the factor.

## Conclusion

In conclusion, results presented in this paper indicate that the ICECAP-A and EQ-5D-3 L are largely measuring two different constructs and thus can be seen as complementary measures, rather than substitutes for one another.

### Ethics compliance

All procedures performed in studies involving human participants were in accordance with the ethical standards of the institutional and/or national research committee and with the 1964 Helsinki declaration and its later amendments or comparable ethical standards. Informed consent was obtained from all individual participants included in the study.
